# Death and Dying in the Emergency Department: A New Model for End‐of‐Life Care

**DOI:** 10.1111/jan.16561

**Published:** 2024-11-11

**Authors:** Kay McCallum, Debra Jackson, Helen Walthall, Helen Aveyard

**Affiliations:** ^1^ Oxford Brookes University, Oxford University Hospitals NHS Foundation Trust Oxford UK; ^2^ University of Sydney Camperdown New South Wales Australia; ^3^ Oxford University Hospitals NHS Foundation Trust, NIHR Oxford Biomedical Research Centre Oxford UK; ^4^ Oxford Brookes University Oxford UK

**Keywords:** death, dying, emergency department, end‐of‐life, end‐of‐life care, patient and family experience, phenomenology, terminal care

## Abstract

**Background:**

Death and dying remain taboo subjects in society today and therefore people may come to the end of their life without having thought about what death and dying might be like and what it is to have a good death. The purpose of this qualitative study was to understand the experience of death and dying in a hospital emergency department. Culturally, some individuals are unprepared for death, and when death occurs in an emergency setting it can be particularly shocking.

**Methods:**

A phenomenological study was undertaken, based on the existential phenomenology of Merleau‐Ponty; and framed by the nurse theorist Hildegard Peplau. Bereaved relatives and registered nurses gave in‐depth interviews describing their experiences and the interviews were analysed using an adaptation of the work of Thomas and Pollio (2002) and Hycner (1985), consistent with Merleau‐Ponty's theories.

**Results:**

The study brings new understanding of what it is like to die in an emergency setting through new understanding of what the accompanying relatives/friends of the deceased person experience, aided by new understanding of the experiences of emergency nurses.

**Conclusions:**

A nursing model based both on the work of Peplau (Parse et al. 2000) and on the work of the nurse theorists Ruland and Moore (1998) and Zaccara et al. (2017) was devised for use in the emergency department when death occurs. It is hoped that this model will help nurses improve the care given to this group of patients and their loved ones.

## 1 Introduction

Care in the emergency department (ED) is focused on protecting and saving life (Bailey, Murphy, and Porock 2011b; Gloss 2017). The skills required to nurse in this acute and fast‐paced environment are highly specialised, and staff work within the framework of the established ED culture (Giles et al. 2019), which is team‐based and time‐dependent (Dawood 2019). This system works well where patients can be promptly assessed, treated and discharged, either at home or in another clinical environment. Such individuals will have a generally positive experience of ED care (Bailey, Murphy, and Porock 2011b). However, some patients present to ED at the end of life and die in the ED. This paper reports on a study designed to explore the experience of end‐of‐life care in the ED, from the perspective of accompanying family members and nursing staff.

## 2 Background

There has, worldwide, for a long time been a drive to improve the care of the dying person, both in hospital and in the community. In general care settings (not emergency care units), certainly in the Western World, the principles of hospice care began to be applied from the 1950s to ‘60s (Quest, Marco, and Derse 2009), and there is evidence that this has transformed end‐of‐life care in general wards (Quest et al. 2012). However, the improvement in care led to a number of scholars, from both nursing and medicine, criticising the current situation regarding the care of dying persons in the emergency department setting (Adamowski et al. 1993; Cauthorne 1975; Jones 1978; Soreff 1979; Ordog 1986), not just in the UK but worldwide (LeBrocq et al. 2003).

For the last 50 years, there have been discussions regarding improving the management of the dying in the ED environment (Cauthorne 1975; Jones 1978; Soreff 1979; Ordog 1986; Adamowski et al. 1993; Edlich and Kubler‐Ross 1992; Tye 1996; del Mar Díaz‐Cortés et al. 2018; Cooper et al. 2018). Recommendations included clear instructions on breaking bad news, supporting the family through their initial reactions and providing support to the multidisciplinary team (Parrish et al. 1987).

Currently, there is no one accepted model of end‐of‐life care within the Emergency Department in the United Kingdom or elsewhere in the world (Weil et al. 2015; Chan 2004, 2006; Chan, Webster, and Bowers 2016; DeVader, Albrecht, and Reiter 2012). Internationally, there is increasing recognition that this is an area of concern. Initiatives such as encouraging closer teamwork are taking place in order to improve care; in the USA (Lamba and Quest 2011; Lamba et al. 2014; Quest et al. 2012; Young et al. 2016), Australia (Weil et al. 2015; LeBrocq et al. 2003) and the UK (Bailey, Murphy, and Porock 2011a, 2011b). There is a conscious drive to include formal palliative and end‐of‐life care education in the medical and nursing curricula in many areas (DeVader, Albrecht, and Reiter 2012; Quest et al. 2012) and initiatives to improve research in this vital area are being developed (Chan 2006; Lamba et al. 2014). Emphasis is being given to prioritising holistic working practices with a focus on viewing individual patient conditions as fluid and constantly changing, and assisting staff to be prepared for these changes (Bailey, Murphy, and Porock 2011c).

However, in order to meaningfully improve practice in this crucial area, there is a need to gain authentic insights into the experiences of death in the ED, from those who are there—loved ones of the deceased person, and the nurses who care for them. The aim of this paper is to report on the findings of a study looking into these authentic insights. This paper is drawn from a larger doctoral study that sought to describe and explain what it was like to experience death in the ED. Previous publications drawn from this work focus on the background of the subject via a literature review and further sought to explore and describe the experiences of research participants who had been bereaved from an ethical and practical viewpoint (McCallum et al. 2018, 2019, 2020).

### 2.1 Methodology

A qualitative phenomenological study was undertaken to look at the experiences people had of being cared for in the ED at the end of life. The particular phenomenological ‘lens’ chosen was that of Merleau‐Ponty, decided upon because his view of the world is exceptionally humane and people‐focused. Merleau‐Ponty saw existence as being in a constant state of awareness of a concept he called the life world. He felt there were four fundamental parts to the life world: body, time, world and others (Merleau‐Ponty 2005). These four parts of the life world correspond to the themes discussed below. Study participants were bereaved relatives and ED nurses. Phenomenology is the investigation of a specific, named phenomenon (in this case, death in the ED), which involves understanding the essence of this lived phenomenon (Wertz 2011) and the experiences of those in a particular situation. These experiences are likely to be shared as storied accounts of lived experience.

## 3 Reflexivity

This research study was undertaken for a PhD degree. The supervisory team were all registered nurses, and the lead investigator was a nurse consultant working with end‐of‐life patients in the ED. None of the participants were known to the team prior to the study. As Olmos‐Vega et al. (2022) mention, reflexivity is rooted in respect for and a valuing of, subjectivity. It can be argued that there are four types of reflexivity, personal (involving ongoing reflection on the part of the researcher), interpersonal (involving collaboration between all members of the research team, ensuring attention to how interpersonal relationships affect the research), methodological (involving constant attention to the way the chosen methodological decisions affect the research process) and contextual reflexivity (referring to how the research questions and the answers obtained are embedded in and influenced by the social and historical context) Olmos‐Vega et al. (2022). In the case of this study, all types of reflexivity were considered and were used constantly throughout the project as seen below. A great strength of the study was the interpersonal reflexivity offered by the research team, all qualified nurses, enabling every aspect of the project to be discussed collaboratively.

### 3.1 Recruitment and Data Collection

We sought to recruit from a variety of sites and therefore participants were recruited via social media. Using social media for recruitment also meant the sample was diverse and included individuals from very different social and cultural backgrounds (Goldman et al. 2023), and there is evidence this method also can help to recruit hard‐to‐reach populations (Darko, Kleib, and Olson 2022). It is acknowledged that social media does also exclude some potential participants. Both participant groups were recruited using a purposive sampling technique.

Phenomenological interviews, interviews which allow the participants to go into depth exploring their feelings about their experiences, were carried out with bereaved relatives (*n* = 11) and ED nursing staff (*n* = 8) by the first author. Refer demographic details in Tables 1 and 2. Relative participants ranged in age from 30 to 73 and the time from the death ranged from 3 to 20 years. Nurse participants were aged from 33 to 55, and all had a minimum of 10‐year experience in the ED. The interviews were to some extent semi‐structured and informed by the quality of death and dying (QoDD) questionnaire, a validated instrument which looks at the quality of the dying experience (Kupeli et al. 2019; Mah, Hales, et al. 2019; Mah, Powell, et al. 2019). The QoDD questionnaire allowed the interviews to flow in a way that focussed on the quality of the experience.

**TABLE 1:** Bereaved relatives participants.

Name * Pseudonym	Transcript number	Approximate age	Length of time since bereavement	Relationship to deceased person
Nicole (P1–1)	1	40s	2 years	Daughter (of mother)
Max (P1–2)	2	40s	3 years	Son (of mother)
Jilly (P1–3)	3	60s	3 years	Daughter (of mother)
Danisha (P1–4)	4	30s	5 years	Wife (of husband)
Bella (P1–5)	5	60s	3 years	Daughter (of father)
Laura (P1–6)	6	60s	2 years	Daughter (of mother)
Pete (P1–7)	7	60s	4 years	Son in law (of mother‐in‐law)
Judy (P1–8)	8	60s	3 years	Daughter (of father)
Carmel (P1–9)	9	50s	15 years	Daughter (of father)
Patricia (P1–10)	10	50s	7 years	Daughter (of mother)
Elizabeth (P1–11)	11	60s	2 years	Wife (of husband)

**TABLE 2:** Nurse participants.

Name * *Pseudonym	Approximate years in practice in the ED	Transcript number	Geographical area
Georgie (P2‐1)	12	1	Manchester
Ed (P2‐2)	8	2	London
Samuel (P2‐3)	10	3	USA
Kate (P2‐4)	4	4	Oxford
Alex (P2‐5)	10	5	London
Andy (P2‐6)	10	6	Newcastle
Sarah (P2‐7)	25	7	Oxford
Martin (P2‐8)	8	8	Oxford

Interviews took place through online means such as Zoom or Microsoft Teams and were carried out individually. Each interview lasted a minimum of 1 h and maximum of 3 h, and there were no repeat interviews.

## 4 Demographic Details

### 4.1 Data Analysis

The interviews were transcribed verbatim and then analysed using a custom tool devised from the work of phenomenological scholars Thomas and Pollio (2002) and Hycner (1985). The transcripts were not checked by the participants. Analysis was initially performed by the first author only, but the whole team participated in all discussions and helped to refine the identification of themes and subthemes.

### 4.2 Rigour

Rigour was considered using the framework devised by de Witt et al. (De Witt and Ploeg 2006). The framework explores five criteria: balanced integration (this refers to the clear articulation of philosophical principles in the context of the topic, the method and the data—hence the discussion about the principles of phenomenology); openness of the study to scrutiny (this emphasises the demand for transparency within the study, hence the need for openness about the aims and purpose of the study); concreteness of issues related to context (this allows the reader to be situated with regard to the data); resonance, or the effect on the reader; and actualisation—the future effects of the research findings. These criteria involve the reader of the research being involved in the findings which should bring them into a sense of the meaning of the findings. In the interests of openness and transparency, all participants were made aware of the background of the researcher and the aims and objectives of the research. The researcher (KMc) also undertook an initial process of reflection regarding their personality and any potential biases. A reflective diary was kept during the research process for the researcher to refer and draw upon.

### 4.3 Ethics

Full ethical approval was granted for the study. The Research Governance Framework (Shaw, Boynton, and Greenhalgh 2005), along with the principles of biomedical ethics (Beauchamp and Childress 2019), was used to ensure ethical rigour. The four principles of biomedical ethics are beneficence, no‐maleficence, autonomy and justice.

Beneficence and autonomy involved safeguarding the participants by ensuring they had all the information necessary to make an informed choice and were completely aware of the implications of taking part in the study. They were also provided with a list of resources for support post‐interview. Confidentiality and anonymity protected the individuality of the participants, ensuring their autonomy. From the justice point of view, the utility of the research was considered (Rubin and Rubin 2011; Flinders 1996), ensuring that the time of the participants was not wasted and assuring them that the study had direct clinical relevance.

## 5 Results

The unique point of the study was the experience of the death of a relative or patient in the emergency department. The results give an insight into the world of the bereaved relatives and nursing staff, giving a snapshot of the minutes and hours they spent with the dying patient in the ED and reveal the complexities and complications caused by the environment they were in. Four themes were revealed through the data. These were: being with others in the ED, the experience of the environment, feelings about the experience and being with the dying person. These themes are elucidated below.

### 5.1 Theme 1: Being With Others

The ED is a large, busy, place. Although some of the participants described being in a single room, and feeling alone, actually, they were surrounded by other patients, families and staff members. Some found it hard being with family, and some found it hard feeling alone in the midst of all the other people. One participant described the stress of coping with his brother as they sat with their dying mother: ‘My stress is about that he's getting edgy and being abrupt and rude with the nursing staff’ (P1‐1(relative)). Another participant said, about the fact that he was with his mother‐in‐law when she died in a single room, with lots of noise going on outside: ‘I think the biggest thing was that I was so alone’(P1‐2/relative).

In all the cases of the nurse participants who were interviewed, the ‘being with’ the families of the dying patients was an important part of their job and they were all aware, both that it was so important and also that it is often not done well. Nursing is rarely a job that is done in isolation, so being with others is a part of any nurses working day. One nurse described how difficult it was when a patient died and nobody had previously brought up the subject of death: ‘Some patients and families haven't had the opportunity to explore what is really important to them, that then gets missed and the patients end up dying before anyone's had the bravery to bring it up. I don't like it at all, I feel like the families will be upset’(P2‐4/nurse).

Nurse participants highlighted the importance of being prepared for death and the difficulties and distress that can arise when these preparatory conversations are not had: ‘people are totally unprepared. And in the ED, we focus on the dying patient so much and it's so quick quick quick … I'm very angry that we don't do it [end of life care] well in the ED. For the relatives particularly’(P2–7/nurse).

Being with others also means the ED staff. Participants expressed an acute awareness that staff in the ED were also emotionally affected by death in the ED: ‘I think we need to remember that end of life care involves not only the patient and the family, but also the staff who are involved and I am really passionate about looking after ourselves as nurses as well’(P2‐8/nurse).

### 5.2 Theme 2: The Experience of the Environment

The participants lived through the hours they describe, being in the world of the ED. Although some of the participants were either healthcare personnel themselves or had attended the ED previously in some other capacity, to all of them, the experience was new and fresh because of the circumstances of that experience. Both the bereaved relatives and the nurse participants described an alien environment, over‐busy, hot and bright. One relative talked about his mother: ‘she stayed in that big noisy place until the end … the whole place was really busy. It seemed to be busy all night’ (P1‐6/relative). Another talked about her feelings immediately after her husband had died: ‘It was all open, I could see people going about their lives and R [her husband] was dead’(P1‐5/relative).

Notwithstanding the general noise and speed and busyness of the ED, relatives greatly appreciated efforts made by ED staff to try to provide privacy and comfort to them. These efforts were experienced as evidence that the ED staff were showing kindness and being caring of them at this difficult time. ‘she was in resus and then they moved her to a quieter area, still within A and E, a single room…they [the staff] got a lovely mattress for us by the side of mums bed, they were so kind.’(P1‐9, relative).

For the staff, the environment was a huge problem when it came to delivering end‐of‐life care. There was a collective sense of powerlessness when they discussed this. One nurse said, of the recent death of a patient: ‘It was no way to die, in a noisy, busy environment, lots of bright lights and people coming and going. I don't think it's right’ (P2‐4/nurse). There is a clear sensation of a lack of control and a feeling that they would love to give good care but are constrained by the nature of their workplace and things they can do nothing about. A nurse said: ‘I really want to be good at this you know, to know that I always give good end of life care. But I can't–every day is so busy, it's all I can do to keep on top of everything. And the layout doesn't help’(P2‐6/nurse).

Nurse participants were also acutely aware that bereaved relatives were also witness to other potentially distressing sights and sounds whilst in the ED. The nurses painted vivid word‐pictures of their experiences: ‘The environment is so important in the ED. It's never ideal … you know, the flow and everything. It would be so great if bereaved relatives did not have to walk past other casualties to see their dead loved one’(P2‐6/nurse).

### 5.3 Theme 3: Feelings About the Experience

The participants all indicated that they were pleased to be able to talk about their experiences, several of them noting that they had not had the opportunity before. Though the participants were being asked to share their stories of a past traumatic event, they wanted to do so to improve the situation for others who may find themselves in the same situation.

‘I don't mind talking about it. I like talking about Mum and I want to help in a small way to improve things for other people’(P1‐1/relative).

In discussing their experiences, the bereaved relatives all described the experience as shocking and distressing, and this included even those whose family members had been very unwell and were expected to die. The fact that this was occurring in ED made the situation more difficult for relatives.

‘I walked [up and down the ED corridor], I didn't know what to do so I more or less just walked … there were lights on, but it just felt dark, it felt strange to me … I just walked up and down the corridor and I just waited’(P1/10/relative).

The relatives experienced a sense of loss of control and described how difficult this was:

‘We saw a doctor a couple of times. Probably. It's hard to tell because they were all in the same sort of scrubs. No introductions. It felt like we couldn't change anything, we didn't know how. Anyway, they only seemed to come and say things were worse. So, we didn't really want to see anybody, we knew it was the end.’(P1‐1/relative).

In sharing their experiences, the nurses revealed themselves as being stoic. They all described that they give everything to their patients every day, they do their best and this includes for patients for whom death is imminent. Nurses did also accept that death is a part of life, and part of life in the everydayness of large and busy EDs. Because of this, nurse participants had come to their own understandings of death.

‘In the ED, some people will die anyway. Some people will improve. And some people we can help to have a good death. Good deaths affect me least because it was good. I think it would help if we as a society could accept that we're all going to die. Death is universal’(P2–4/nurse).

This is not to say that staff don't get upset at a death, and nurse participants described feeling an emotional reaction to the death of every patient and that this feeling of grief continued, regardless of years of experience:

‘It [the grief] never goes away. It always always affects me. I couldn't do my job if it didn't. I don't always remember everyone's names. But I do remember my patients and I grieve for those who don't make it.’(P2–7/nurse).

Nurse participants highlighted the emotional toll of grief, and recognised the importance of taking the time to grieve for each patient, ‘We don't get time to grieve and that's important because we do grieve’(P2‐7/nurse).

### 5.4 Theme 4: Being With the Dying Person

The participants described exactly what it was like for them to be witnessing end‐of‐life symptoms in such a busy environment. One relative recalled her experience and the desperation she felt to get help and support for her family member:

‘she was in pain, crying with pain in her abdomen … I asked a nurse for some pain killers and it took an age to get anything … [the doctor said] he thought the situation was very serious, she was really sick, she was vomiting by that stage … I felt really desperate’(P1‐11/relative).

Participants generally had held onto some extremely vivid memories, that had remained with them over time: ‘I had a very intense time with her in accident and emergency you know … the memories of my mum were all about when she was ill, and at the end, the smell of her hair, her unwashed head and all that … my visual memory has all been erased of my mum as a healthy person’(P1–4/relative).

The nurse participants had equally vivid memories: ‘He was probably dead, on arrival at the hospital, but we treated him as if there was a chance ‐ rushed him into resus and started all the procedures…we worked on him for ages and we didn't get him back and we were not surprised. His wife and young son were outside and I had to go and tell them and my god it was horrible. I took them into an empty side room, and she said, he's gone, hasn't he? And I said he has, and I am so sorry, I wanted to let you know that we really tried. Really really tried’(P2–5/nurse).

Though the nurses reported being emotionally affected by the loss, they kept their focus on immediate care of the person after death, and to providing emotional support and care to the family.

‘[when the person has died] there were three people in this room and now there are two. The person is gone, couldn't be saved, and it affects everyone, but as a nurse, your job is not finished, not only do you have the last offices to do, you also have the family to look after’(P2–7/nurse).

After the death, there was also a sense of lack of closure that was distressing to all parties. One participant said:

‘That was it. I did go to see her, to see her dead body, but when it came to it, I felt I couldn't go in the room, and I just left the building and sat in the car. And at that point my engagement with the ED finished’(P1‐1/relative).

Another, who felt she had had minimal engagement with the staff, said:

‘…even after I was told he had passed you know, that was the end. There was nothing else. That was it. So final’(P1‐6/relative).

Nurses also felt this lack of closure after a death. Often this is related to the busy and chaotic environment:

‘I've had to transfer patients along busy corridors full of people – patients who didn't look like they were going to survive for much longer. There was no privacy. I refused once and it was awful – I said I'm not moving that patient she's going to die any minute. Even though it was so chaotic in the department’ It's like that after a death, no chance to reflect’(P2‐7/nurse).

Both sets of participants thus described feeling out of control and distressed following death in the ED.

### 5.5 Limitations

Limitations of the study relate to the search strategies undertaken, the heterogeneity of the studies identified (including several from the same data set) and limitations of the quality framework used. There is also the potential for language bias (all the studies were in English) and publishing bias (publications post‐1990 only).

## 6 Discussion

The data from the participants confirms that care in the ED is underpinned by the universal biomedical model, a model which focusses purely on the suffering body, reducing this body to a collection of cellular abnormalities (Johansson 1998). The purpose of the ED is to save lives. People come into the ED as broken, hurt, bodies and the expectation is that the ED will fix these problems (Cypress 2014; McGuinness 1988; Chan 2011). The care is very fast‐paced, technology‐dependent and goal‐focussed. Death is not denied, but the preference is that it belongs somewhere else. The staff operate under pressure to quickly move the patients through and find solutions and all within a 4 h period (in the UK) before the patient ‘breaches’ guidelines, and the health service is liable to be subjected to a financial penalty (Glen, Constanti, and Brohi 2016). This model of care in the ED works well according to the literature (Cooper and Grant 2009; Calvello et al. 2013) and ensures safe practice, with staff working within hierarchies and holding known, established roles (Hassan 2018). In the UK where this study was conducted, the top of the hierarchy is the ED consultant who is the ultimate accountable decision‐maker in relation to the treatment plan for each patient (http://www.rcem.ac.uk, 2015).

However, care of the physical body as per the biomedical model, whilst important particularly in settings such as the ED, is not enough. Despite the view of the body as a machine, object or thing, it is important to note that to a patient it is never experienced in this way. To a patient their body is everything, it is far more complicated than a machine, an object or a thing. The nursing profession has had for many years a specific focus on holistic care (Papathanasiou, Sklavou, and Kourkouta 2013; Brooker and Waugh 2013); nursing assessments are therefore centred on the care of the entirety of what a person is (Thomas and Pollio 2002). The phenomenologist Merleau‐Ponty, whilst acknowledging that we are our bodies in the most earthy way—we weep, we eat, we excrete—nevertheless spent his career emphasising that we are much more than this, we are sacred, whole and complex beings (Merleau‐Ponty 2005).

Unfortunately, the environment of the ED is such that the dominance of the biomedical model can make it difficult for nurses to demonstrate a uniquely nursing contribution. Holistic nursing care, whilst valued by staff, is overshadowed by the dominant biomedical culture, and not valued enough. In the very medically dominant world of the ED, uniquely (in healthcare) constrained by time factors and the need to save lives, nurses are having, in looking after dying patients who cannot be saved, to move between two paradigms—the caring, holistic nursing‐values driven model and the biomedical model. It is questionable whether this is even possible; the nurses who participated in this study adhered to the biomedical model, whilst acknowledging the holistic model, and expressing they were unable to practice the holistic model due to the work cultures they were operating in.

It is generally globally acknowledged that end‐of‐life care in the ED has room for improvement. One solution has been to increase the presence of palliative care clinicians in the ED alongside improving the education given to ED clinicians regarding palliative care (del Mar Díaz‐Cortés et al. 2018; Gloss 2017; Gisondi 2009; http://www.rcem.ac.uk, 2015). This, although an apparently straightforward and elegant solution, has been worked on for several years, and there is little evidence that it has actually improved care of the dying though it has undoubtedly increased the knowledge of clinicians working in the ED around the principles of palliative care (Meo, Hwang, and Morrison 2011; Mierendorf and Gidvani 2014).

A problem with this approach is that palliative care in the ED also conforms to the biomedical model. Medical practitioners naturally continue to take decisions about what is best for their dying patients—in some cases, for example, complex symptom management, this is of course highly appropriate— (Fukuzawa and Kondo 2017; Heyland et al. 2006; Clark 2002), and there is acknowledgement that palliative care is not exempt from this medicalisation of death (Clark 2002). Participants who took part in this study all referred to the lack of control and lack of involvement in the care of the patient; how they were separated from care delivery. Fear of loss of control may be the most important concern for patients at the end of their lives and for their families (Caruso‐Herman 1989; Borgsteede et al. 2006; Holdsworth 2015; Mah, Hales, et al. 2019). Allowing and promoting control at the end of life is essential for the patient to have a good death; decision‐making in palliative care therefore needs to be with the patient and not for the patient (Winnington, Holroyd, and Zambas 2018). Palliative care, even in the hospice setting, continues to be under the jurisdiction of medical professionals (Lynch et al. 2011), which may prevent the patient and family from being involved in decisions about care, thus perpetuating the biomedical model right to the very end of life. The participants in this study certainly did not feel they had any control as their relative or in fact their patient approached the end of life. The nurses felt powerless and unable to change anything and the relatives felt that everything that was happening was out of their control. Nurses have an essential role at the end of life, and nurses need to be supported and enabled to carry out this essential role in the ED. Palliative nurses are fortunate in that they can nurse in a truly holistic way, at least in palliative settings (Lynch et al. 2011). But palliative nurses do not work in the ED. ED nurses, as demonstrated in the data, work within the biomedical‐model culture of the ED and are not empowered to care in a way that promotes a good death in such an environment.

## 7 Recommendations

### 7.1 The Creation of a New Nursing Theory‐Focused Model

**FIGURE 1:**
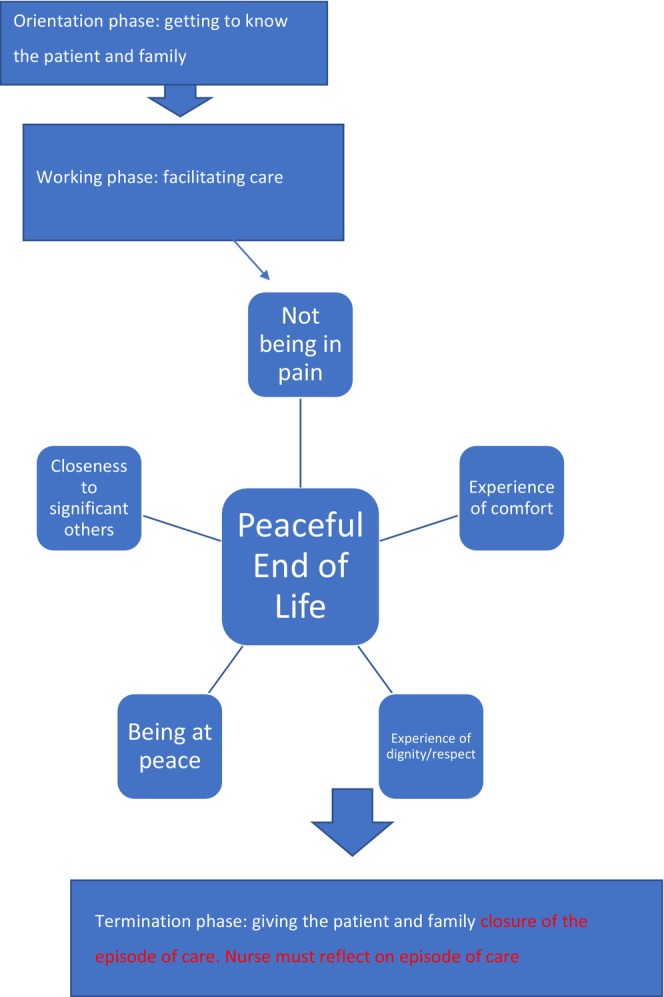
A new nursing model for end‐of‐life care in the ED.

Using the data gained from this study and combining the work of Hildegard Peplau with the peaceful end‐of‐life theory (Ruland and Moore 1998), a new model for end‐of‐life care in the ED was devised. The model is shown in Figure 1. The model was designed to be as easy and simplistic to use as possible, to ensure it was appealing to nurses working in a busy and stressful ED environment.

### 7.2 Hildegard Peplau

In the mid‐twentieth century, the nurse scholar Hildegard Peplau, influenced by phenomenology (Brunero, Jeon, and Foster 2015), developed a deductive theory of nursing which she called the interpersonal relations model (Peplau 1997). The model innovatively moved away from the idea that nursing meant assisting the medical staff; Peplau felt that the nurse instead should work with (alongside) the doctors and other health professionals to promote the patients' well‐being (Gastmans 1998). The model explains the nurse–patient relationship as developing through four overlapping stages: orientation, identification, exploitation and resolution or termination (closure) (Peplau 1997). It is assumed that a patient is someone who has something wrong; the orientation phase takes place when the patient accepts this and seeks help from the nurse. The identification phase is about the patient selectively working with the people who can offer help, solutions to the identified problem are sought and the therapeutic relationship grows, with the nurse deliberately using his or her skills to identify with the problems of the patient and understand their unique experience. The exploitation phase is concerned with the patients' use of the nurse–patient relationship amongst other nurse‐identified resources to move forward towards resolution, and the resolution/termination/closure phase results from the successful completion of the other phases—the nurse–patient relationship can then be dissolved, and the patient can move forward (Peplau 1997; Gastmans 1998; McCrae 2012). In many cases sadly, the moving forward element means moving forward to the end of life. Peplau's emphasis on patient experience makes her model an example of the practical use of phenomenology in nursing and in nursing research.

### 7.3 The Peaceful End‐of‐Life Theory

The theory of the peaceful end of life is a mid‐range nursing theory which was developed in the 1990s (Ruland and Moore 1998). Its authors, both practising nurses, sought to address the perennial problem of the theory‐practice gap in nursing by developing a theory from an unpublished set of standards of care for the dying patient in an acute setting (Ruland and Moore 1998). They chose to develop this as a mid‐range theory to ensure it was succinct, brief, and easily usable by practising nurses, unlike the original standards of care document, which was too detailed, unstructured, and unable to be rigorously tested. The theory involves five concepts—not being in pain, experience of comfort, experience of dignity/respect, being at peace, and closeness to significant others/persons who care. The theory of the peaceful end of life depends on the nurse proactively acting as a facilitator to enable the patient to gain benefit from all concepts of the theory. Peplau's interpersonal relations theory aligns well with the peaceful end of life theory, if, during the ‘working’ phase of Peplau's theory, the nurse proactively works with the patient to ensure the five concepts are addressed.

End‐of‐life care is difficult in such a high‐pressured environment; the above model gives nurses a succinct framework through which to practice. This model attempts to show how the theory of the peaceful end of life aligns with Peplau's theory of interpersonal relations to improve care of the dying person in the ED environment. The key lies in excellent communication between the patient/family and the nurse and a commitment to the nurse proactively working with the patient and family to facilitate all five of the elements of the peaceful end‐of‐life theory. The nurse accepts responsibility for ensuring that attention is paid to all elements and, as per Peplau's theory, uses self‐awareness and reflection at all stages. Care thus becomes fully patient/family focussed and simultaneously the nurse is empowered to work ‘with’ the patient/family rather than doing things to/for the patient and family. This approach may bring better satisfaction with the care episode for all concerned. The model above showing the integration of the peaceful end‐of‐life theory with Peplau's theory of interpersonal relations could also help with the unique problem in ED of the nurse not having much time to establish a caring relationship with the patient and family.

### 7.4 Conclusion

In this study, we phenomenologically examined the experiences of those who were with their loved ones at the point of death in the ED and the nurses who look after these patients. Analysis of the storied accounts reveals the complexity of experiences for those witnessing death in the ED, and the nature of the memories of the experience that were retained synthesis of the findings with extant theory and literature led to the creation of the ED end‐of‐life care model which aims to provide a peaceful death for patients and also meet the neds of friends/relatives and nursing staff in the ED. Future work is planned to test this model in practice.

## Conflicts of Interest

The authors declare no conflicts of interest.

## Peer Review

The peer review history for this article is available at https://www.webofscience.com/api/gateway/wos/peer‐review/10.1111/jan.16561.

## Supporting information


Data S1.

## Data Availability

The data that support the findings of this study are available on request from the corresponding author. The data are not publicly available due to privacy or ethical restrictions.
